# Exploring Empowerment in Online Support Communities for People Living With Tic Disorders and Tourette Syndrome: Qualitative Survey Study of User Experiences

**DOI:** 10.2196/66912

**Published:** 2025-10-09

**Authors:** Ella C Ford, Neil S Coulson, E Bethan Davies

**Affiliations:** 1 Lifespan and Population Health School of Medicine The University of Nottingham Nottingam United Kingdom; 2 School of Humanities and Social Sciences Leeds Beckett Univeristy Leeds United Kingdom; 3 NIHR MindTech HealthTech Research Centre Institute of Mental Health School of Medicine, The University of Nottingham Nottingham United Kingdom; 4 Clinical Neurosciences and Mental Health Institute of Mental Health School of Medicine, The University of Nottingham Nottingham United Kingdom

**Keywords:** empowerment, online health communities, Tourette syndrome, qualitative research, web-based survey, support community, tic disorder, Tourette, survey, peer support, emotional support, mental health

## Abstract

**Background:**

People with tic disorders (TDs)—such as Tourette syndrome—report poorer quality of life compared to non-TD peers, and experience considerable difficulties, including societal stigmatization and barriers to accessing health care and evidence-based interventions. Peer support can help improve psychological outcomes, and online support communities (OSCs) are one way to access psychological support. Empowerment involves improving an individual’s cognitive processes to increase their ability to assert control over their health condition. OSCs have been suggested to facilitate empowerment, but this has not yet been investigated in users of OSCs for TDs.

**Objective:**

This study aimed to explore empowerment processes and outcomes present in OSCs as perceived and reported by users of OSCs for TDs living with a TD.

**Methods:**

A web-based survey of current users of OSCs for TDs (n=39) was conducted in summer 2022. The survey included four free-text questions about the impact of using OSCs for TDs in relation to empowerment, such as how it has affected their interactions with health care professionals (HCPs), decisions about treatment, and their experiences of living with a TD. Survey responses were analyzed using deductive and inductive reflexive thematic analysis guided by an initial coding structure derived from the empowering processes and outcomes theoretical framework.

**Results:**

Analysis of responses identified a range of empowering processes (eg, exchanging information, encountering emotional support, finding recognition, and sharing experiences) and outcomes (eg, being better informed, feeling more confident in the relationship with HCPs and in making treatment decisions, and enhanced well-being) as related to OSC use in people with TDs that were consistent with the theoretical framework. Additionally, the process of changing stereotypes and stigma, the outcomes of raising awareness, and “one size does not fit all” were identified. A small number of disempowering processes and outcomes were identified, notably the outcome of feeling less confident in the relationship with HCPs.

**Conclusions:**

These findings contribute to evidence for empowerment processes and outcomes experienced by users of health OSCs generally and highlight unique aspects of empowerment for users of OSCs with TDs and how these affect their experiences. OSCs appear to be an important tool in improving mental well-being through validation and recognition from peers, related to the acknowledgment of feelings of imposterism. Using OSCs can expand knowledge through exchanging information and experiences they may not have otherwise had access to—increasing empowerment through improvements in self-management and confidence in treatment decisions. However, this can also decrease trust in HCPs and negatively impact relationships due to decreased hope of assistance and fear of stigmatization for using OSCs. The authors suggest that recommendations regarding the use of OSCs are presented in self-management guidelines concerning coping strategies and the importance of peer support in living with tics.

## Introduction

Tic disorders (TDs)—such as Tourette syndrome (TS) and chronic tic disorder (CTD)—are neurodevelopmental conditions characterized by tics: rapid, persistent, involuntary movements or vocalizations [1]. TDs are more common in men than women [2], and symptoms tend to have their onset during childhood. If tics are present for more than 12 months, assessment for CTD (presence of motor or vocal tics) or TS (motor and vocal tics) can be considered. Global prevalence of these TDs is estimated to be 0.69% (vocal CTD), 1.65% (motor CTD), and between 0.50% and 0.77% (TS) [2,3]. Tics typically wax and wane in frequency, severity, and impact over time, with variable prognosis as individuals age [4,5]. For most people living with TDs, tics normally decline through adolescence and early adulthood, with remission possible—however, for others, tics continue into adulthood [4,5]. TDs are incurable, but evidence-based pharmacological and behavioral interventions can aid symptoms [6]. However, people with TDs consistently report difficulties accessing health care and appropriate interventions, including a perceived lack of awareness among health care professionals (HCPs) and a scarcity of available treatment options [7-9].

There are many physical, psychological, societal, and cultural challenges associated with living with tics. Compared to adults without TDs, those living with TDs report a lower perceived quality of life, heightened levels of psychological difficulties [10-12], and are more likely to attempt and die by suicide [13]. TDs, in particular TS, are widely misunderstood and stigmatized in society; people with TDs experience many stigmatizing consequences, including anticipated and actual discrimination, bullying, mistreatment, and ostracization [14]. Tics are often visible and witnessed by others, frequently resulting in unwanted attention and negative comments [15]. Although tics are involuntary, many people with TDs report being able to delay or suppress their tics [16] to “fit in” and avoid stigmatizing responses [17-22]. However, this can be painful [9] and may strengthen self-stereotyping beliefs about having TDs, such as feelings of inauthenticity around “faking” tics [14,17]. These experiences of interpersonal, community-level, and structural stigmatization can negatively impact an individual’s attitudes about their tics and lead to avoidance of others and social isolation [14].

Peer support may help mitigate the social exclusion that people living with TDs frequently report [10,23]. As theorized by the direct effects hypothesis of social support [24], peer support can help ease isolation and loneliness and improve psychological outcomes through fostering connections. Peer-led support groups typically involve sharing and receiving several facets of social support, such as emotional, appraisal, and informational support, with this support being based on experiential knowledge rather than that obtained from HCPs [25]. The expansion and increased access to internet-enabled technologies in everyday life have led to the creation of asynchronous text-based online support communities (OSCs) for those living with specific health issues, wherein individuals can access peer support [26]. Text-based OSCs are unique as they can be accessed regardless of time and location, unlike physical support groups [27], and often provide access to a backlog of user discussions, as well as the ability to actively engage by posting new content and engaging with content posted by other members. There has been considerable growth in the number of OSCs available to individuals living with long-term conditions (LTCs) in recent years [28,29]. Research regarding the impact of OSCs has been conducted with people living with a range of LTCs such as HIV, diabetes, and arthritis [29-31]. A web-based survey of users of OSCs for TDs—including people with TDs and caregivers—distinguished multiple mechanisms through which users and caregivers benefit from membership [32]. This included improved knowledge of their condition and common co-occurring issues, increased confidence and understanding around communication with HCPs, positive impacts on their psychological well-being and appraisal of having tics, and an increased sense of belonging. These benefits also appear to relate to empowering processes and outcomes.

Empowerment and social support are interconnected concepts, but have some important distinctions. Social support is generally theorized as the perception of care, assistance, and comfort from a defined social network with functions such as emotional and informational support and companionship [33]. Empowerment, on the other hand, is a complex, multifaceted concept that functions to improve a person’s ability to be more in control of their own life and their power in decision-making [34]. It is concerned with an individual’s cognitive processes around increasing their autonomy and decreasing powerlessness, increasing their awareness of and building their strengths [35]. Empowerment has been hypothesized to be facilitated through engagement with OSCs, with several potential positive outcomes for people living with LTCs, including protecting members’ self-identity, improving the self-management of their condition, and developing their confidence in their ability to improve relationships with HCPs [34,36,37]. The processes through which these outcomes are gained include social support elements, such as supporting and being supported by peers with their condition, finding recognition, and becoming better informed through the sharing of knowledge [30,34,36]. Though increasing support is an important facet to OSCs, they also act as a complement to health care expertise—enabling individuals to translate this first-hand experience into practical self-management techniques, expanding their knowledge whilst providing informational, emotional, and social support [34]. This knowledge empowers individuals to have responsibility over their health and to make independent management decisions [38]. In short, this knowledge development is considered separate from social support, but this knowledge may be acquired through it [39]. The type and frequency of interactions with OSCs can differ [40] and have also been suggested to affect the presence of empowerment and associated outcomes in some, but not all, communities [41-43]. As well as at the individual level, empowerment can also occur collectively within OSCs, wherein individuals engage as a group to increase their social power and create change for their condition, such as through policy reform [44-46]. Collective empowerment has important potential implications, such as building a collective “voice” for action and subsequently improving support services and societal attitudes [22,45].

Recent literature suggests that empowerment may be a useful framework to explore the relationship between engagement in OSCs and improved psychosocial outcomes [34,44,47]. Increased perceptions of one’s empowerment have been associated with several health-related outcomes, including improved quality of life and reduced condition-related impairment [31,48,49]. As OSCs have the potential to improve empowerment and psychosocial outcomes for individuals with TDs, they could be a useful tool to complement HCP care and aid self-management [34,50]. To date, research exploring empowerment processes and outcomes in individuals with TDs is limited: previous findings suggest OSCs are a space where these can be fostered and result in “offline” outcomes such as improved communication with HCPs and increased well-being [32,51]. Therefore, this study aimed to explore empowerment processes and outcomes present in OSCs as perceived and reported by users of OSCs for TDs to understand how OSC use may affect empowerment, particularly in relation to health care interactions and self-management.

## Methods

### Study Design

This cross-sectional study consisted of a web-based survey completed by current users of OSCs for TDs. A web-based survey approach was chosen as it allowed a “wide-angle” lens on the topic due to the ability to recruit a larger number of participants, as compared to more time-intensive qualitative approaches, from geographically dispersed locations, increasing the diversity of voices [52]. This approach may also appeal more to people living with stigmatized conditions such as TDs due to their felt anonymity [53].

A qualitative phenomenological approach within a constructivist research paradigm was appropriate for this study, as it focused on understanding participants’ experiences and perceptions of participating in OSCs, enriched through broader social contexts and integration of existing theory [54]. The analysis was guided by the theoretical framework of empowerment in OSCs developed by van Uden-Kraan et al [36,55].

This study is reported in accordance with the SRQR (Standards for Reporting Qualitative Research; Multimedia Appendix 1) [56], the CHERRIES (Checklist for Reporting Results of Internet E-Surveys; Multimedia Appendix 2) [57], and the CROSS (Checklist for Reporting of Survey Studies; Multimedia Appendix 3) [58].

### Participants and Recruitment

Recruitment took place over 6 weeks from June to July 2022. To be eligible for the study, participants needed to be aged 16 years or older. As the purpose was to investigate experiences of OSC use of people living with a TD, participants needed to be current users of at least one OSC for TDs and have a self-reported confirmed or suspected TD diagnosis or symptoms of a TD. A formal diagnosis was not required to participate due to the aforementioned barriers to health care, which include assessment and diagnosis [59,60]. Akin to the previous approach taken by authors [32], studies were advertised through charities for TDs (eg, Tourette’s Action) and primarily placed directly on OSCs, specifically inclusive of or exclusively aimed at people living with TS or TDs. These OSCs were identified by using a mix of key terms (eg, “Tourette,” “tics,” and “support”) across Facebook, Reddit, and Discord social networks or platforms. To be approached to share the study, the OSC needed to operate predominantly in English, allow advertisements of research studies to be posted or shared on the OSC, have had activity within the past 30 days (eg, users posting messages), and have ≥100 members [61]. Previous studies have used similar criteria to identify actively used OSCs to reach current users [49,62]. Both open (ie, no membership required to access OSC) and closed or private OSCs were targeted for recruitment. In total, 26 eligible OSCs were identified: four were public, and the study advert was posted directly onto the OSC. For the 22 closed or private OSCs, the first author (ECF) directly messaged the OSC’s moderators to request permission to post the advert to the OSC: 13 moderators responded and provided permission to post the study to their OSC. In total, the study advert was shared on 17 distinct OSCs. Based on a previous similar study of the same population group, the target sample size was 76 participants [32]. Data collection ended in July 2022 due to time restrictions, as the research was conducted as part of a postgraduate degree.

### Ethical Considerations

This study was reviewed and received ethical approval by the MSc Health Psychology ethics review panel at the University of Nottingham (MEDS4008-22-15) and was conducted in accordance with the ethical standards of the Declaration of Helsinki. All OSCs allowed advertisements of research studies to be shared, and permissions were sought and granted from moderators to advertise the study in closed or private OSCs. Participation was voluntary, and informed consent was obtained from all participants electronically through the web-based survey. Participants were not asked to provide personally identifiable information. Data collected was anonymous, deidentified, and stored securely. TDs are suggestible; talking, hearing, or reading about tics is known to increase premonitory urges and tics themselves [63]. Therefore, this potential risk was clearly stated in the participant information sheet, and prior to the open-ended questions. Participants were informed that they could withdraw from the study at any time should they become uncomfortable. To further minimize potential distress, the participant information sheet and debrief contained a list of TS or TD organizations and crisis hotlines operating worldwide. No compensation was provided for participants.

### Procedure

All advertisements provided brief information about the study and a hyperlink to the open web-based survey (hosted on Jisc Online Surveys), which took approximately 20 minutes to complete. Prior to going live, the survey was piloted by a person with a TD to ensure the questions were acceptable and understandable, to assess the usability and technical functionality of the survey, and to estimate the time to complete—with relevant changes undertaken in response to their feedback (eg, consolidating two items about passive and active use into one Likert scale measuring passive to active use). The survey consisted of 11 pages: the first 2 web pages consisted of information about the study, including the estimated length of time of the survey, information regarding data storage, investigator details, and the purpose of the study, followed by a consent form on the third page in which participants provided informed consent through selecting several mandatory tick-boxes. The next 7 pages consisted of the survey questions: first, participants answered several demographic questions about themselves (gender, age, and country of residence), whether they had a confirmed TD diagnosis, self-reported co-occurring conditions, and use of medication and behavioral therapies for TDs. Tic-related impairment was assessed through 2 items from the impairment subscale of the Yale Global Tic Severity Scale—self-report version [10]. The Online Peer Support Group Use Scale [64] was used to collect information regarding participants’ use of OSCs for TDs, including how many OSCs for TDs they were members of, how long and how often they used OSCs, their satisfaction with using them, and their estimated use on a scale of passive-active engagement in the past week. Active use was defined as direct use, such as posting content, replying to posts, and commenting on others’ responses; passive use was defined as indirect use, such as browsing, reading, viewing, and “liking” posts. Finally, participants were presented with 4 open-ended questions (Textbox 1; adapted from Holbrey and Coulson [31]) relating to participants’ use of OSCs for TDs and how they may relate to empowerment. The final page debriefed participants. All items except open-ended questions were required to be completed to proceed with the survey. All demographic items had a “prefer not to say” option, except for the TD diagnostic question, as this was required to assess questionnaire eligibility. Participants could go back and revise responses if needed, and could provide their email to receive a link to come back to the survey later. This is automatically provided by Jisc Online Surveys; researchers did not have access to participants’ email addresses. All participants were allocated a unique response number by Jisc Online Surveys.

**Open-ended questions used in the web-based survey.**

- How has your use of online support communities (OSCs) affected your interactions with health care professionals about Tourette syndrome (TS)/tic disorders (TDs)/tics, if at all?
- How has your use of OSCs affected your decisions about treatment and management for your TS/TDs/tics, if at all?
- Can you explain if using the OSC has changed your perceptions and experience of living with TS/TD/tics?
- Do you have any further comments about your experiences using the OSC for TS/TDs/tics?

### Data Analysis

Descriptive statistics were calculated, including the absolute and relative frequencies of categorical variables and the mean and SD of continuous variables. A reflexive thematic analytical approach, as outlined by Braun and Clarke [65], was used to analyze the qualitative responses to the 4 open-ended questions with a predominantly deductive orientation to data coding, involving several stages of familiarity, coding, and grouping codes. The authors (ECF and EBD) jointly created an initial coding framework based on the empowering processes and outcomes questionnaires and theoretical framework developed by van Uden-Kraan et al [36,55] (Multimedia Appendix 4).

This framework outlines seven overarching consequences of engagement with OSCs (eg, increased confidence in the doctor-patient relationship and enhanced social well-being) and five processes (eg, exchanging of information and encountering emotional support) through which these consequences occur [55]. A similar framework was used to analyze experiences of using OSCs for HIV [47] and prostate cancer [66]; therefore, this deductive orientation was considered useful to provide a lens through which to interpret the data and provide more depth to previous literature on empowerment within OSCs. Minor changes were made to the original wording of van Uden-Kraan et al [36] of some empowerment elements in the framework, such as changing references from “physician” to “health care professional,” and references to “illness” to “condition” to reflect terminology more appropriate to TDs. In line with Braun and Clarke’s [65] suggestions in conducting deductive thematic analysis, the authors worked from a “curious, open, and questioning” position. Throughout the analysis, authors reflected on the “fit” of the framework with the data, ensuring this was not limiting the interpretation of the data, exploring both how the framework was evidenced and how it was not, and in what ways. The authors developed and refined the codebook throughout the process and used inductive techniques in identifying and coding data that did not appear to map onto the framework.

Participants’ responses were first anonymized and uploaded into the software NVivo (version 14; Lumivero). The first author (ECF) conducted the first two stages of analysis. This involved familiarization of the data through reading and rereading of participants’ responses. The data were then coded using the predefined empowerment outcomes and processes coding framework. Several samples of data were cross-checked by a second researcher (EBD). Data that did not appear to map onto these a priori codes were identified, reflected on, and discussed between the two researchers alongside the coding framework overall. Where the terminology used for processes and outcomes in the predefined framework appeared not to completely align with what was found in the data, minor adjustments to the labels within the coding framework were made. First, the outcome “feeling more confident about treatment” was changed to “feeling more confident about treatment decisions,” to reflect that this included decisions not to have treatment. Second, the word “optimism” was removed from the phrasing of the “increased optimism and control over the future” code, as evidence of increased optimism was not found in participants’ responses. Third, there appeared to be a conceptual overlap in the “enhanced self-esteem” and “improved acceptance of the condition” codes: these codes were combined, along with references to mental well-being, as there was considerable overlap between these concepts. This resulted in the “enhanced acceptance, self-esteem, and mental well-being” code. Negative aspects relating to some of the overarching codes were also found in the data. These were negatively coded from their positive counterpart (eg, “decreased confidence in the relationship with HCPs,” as a counterpart to “increased confidence in the relationship with HCPs”) and embedded in the results for the overall theme. Additionally, through in-depth discussion, the authors developed and assigned inductive codes to the data that did not appear to map onto this predefined coding framework, resulting in some themes outside of the original framework. The first author had previously performed an inductive thematic analysis of the data—the codes and themes derived from this were compared with those of the present analysis and found to be similar.

### Positionality Statement

This study was part of ECF’s MSc Health Psychology dissertation, supervised by other authors EBD and NSC. ECF has experience working in health care services. EBD has research expertise in the field of TDs and NSC in the field of OSCs. Both EBD and NSC have previously conducted research around empowerment, as well as OSCs for people living with TDs, and all authors have experience conducting qualitative survey research. No authors have lived experience of TDs, and therefore are outsiders to the topic; however, all authors have experience of using health OSCs. Authors consulted with a person with lived experience of TDs in the development of the survey and incorporated their input.

## Results

### Descriptive Statistics

A total of 58 participants responded to the web-based survey. Of these, 39 participants provided free-text answers to at least one (of the four) open-ended questions and were included in the analysis. This was markedly lower than the targeted sample size, potentially due to the optional nature of the open-ended questions and the limited time available to recruit, which may have limited the range of perspectives captured. Participants were mainly female (19/39, 49%), based in the United Kingdom (20/39, 51%), and had received a TD diagnosis (29/39, 74%; Table 1). The majority accessed an OSC for TDs once (10/39, 26%) or twice (8/39, 21%) daily, with 13 (33%) participants checking between three to five times daily (Table 1). Participants estimated that a mean of 46% of their time using OSCs was “active” use as opposed to “passive” use in the last week.

**Table 1:** Demographic makeup of the sample and their self-reported use of OSCs^a^ for TDs^b^.

Characteristic		Participants (n=39)
Age (years), mean (SD)		25.5 (14.2)
**Sex, n (%)**		
	Female	19 (49)
	Male	8 (21)
	Nonbinary	10 (26)
	Other^c^	2 (5)
**Country of residence, n (%)**		
	United Kingdom	20 (51)
	United States	11 (28)
	Australia	4 (10)
	Canada	2 (5)
	Egypt	1 (3)
	Italy	1 (3)
**Diagnosis status, n (%)**		
	Diagnosed with a TD	29 (74)
	TD symptoms and currently awaiting assessment	7 (18)
	TD symptoms and currently not waiting for assessment	2 (5)
	Prefer not to answer	1 (3)
**Taking medication for TDs, n (%)**		
	Yes	9 (23)
	No	30 (77)
**Received behavioral therapy for TDs, n (%)**		
	Yes	7 (18)
	No	31 (80)
**Self-reported co-occurring conditions, n (%)**		
	Attention-deficit/hyperactivity disorder	13 (33)
	Autism	9 (23)
	Obsessive compulsive disorder	19 (49)
	Anxiety	22 (56)
	Depression	17 (44)
	Functional neurological disorder	4 (10)
	Other	5 (13)
**Number of different OSC memberships, n (%)**		
	1	7 (18)
	2	12 (31)
	3	8 (21)
	4	4 (10)
	≥5	8 (20)
**Reported length of OSCs membership, n (%)**		
	1-6 months	6 (15)
	6-12 months	14 (36)
	1-2 years	9 (23)
	≥2 years	10 (26)
**Reported daily use of OSCs, n (%)**		
	1 time	10 (26)
	2 times	8 (21)
	3 times	4 (10)
	4 times	4 (10)
	5 times	5 (13)
	≥ 6 times	8 (21)

^a^OSC: online support community.

^b^TD: tic disorder.

^c^Other sexes indicated in free text response were “trans man” (n=1) and “two spirit” (n=1).

### Evaluation Outcomes

#### Overview

Analysis of participants’ responses to the open-ended questions resulted in the following themes, which mapped onto the empowerment processes and outcomes framework used for analysis, as well as arising additional inductive themes. Negative aspects affecting these processes and outcomes are also highlighted and discussed. Quotes are provided verbatim.

#### Empowering Processes Within OSCs

##### Exchanging Information

Participants described OSCs as being reliable places to ask questions and exchange valuable information regarding living with TDs, as well as for signposting to resources for further support:

I feel like now I have a community that I know who to go to if I have problems and so they know who to avoid and how to deal with things.

P16, 16 y, other

They found the information provided in OSCs to be particularly relevant and accessible, compared to knowledge acquired elsewhere:

It’s also been particularly useful to see how Tourette’s [sic] can coincide with other disorders like ADHD [Attention Deficit Hyperactivity Disorder], as that kind of information is much harder to find in things like medical studies or articles.

P37, 17 y, nonbinary

For others, the information provided a substitute for accessing HCPs, as they were able to learn about their condition and self-management strategies whilst waiting for care:

I don’t have access to professional treatment currently due to waiting lists, so I’ve learned tic redirection techniques from other people online.

P7, 17 y, nonbinary

However, some participants complained about the lack of information organization; one participant noted their misinformation concerns:

As someone working in Healthcare [sic], I do see a huge amount of misinformation.

P28, 22 y, female

##### Encountering Emotional Support

Participants placed considerable importance and appreciation on being supported by their peers, often over other sources of support:

The people online have treated me better then [sic] my own family, and they’ve supported me better then [sic] my family, they saved my life.

P17, 16 y, other

They valued the opportunity to be supported by people who shared their condition:

They are a great resource, especially for people who don’t know anyone with tics in real life and don’t have a lot of support.

P36, 16 y, female

Much of the emotional support described was in the form of reassurance relating to feelings of “imposter syndrome” such as being fraudulent:

I have imposter syndrome surrounding my disorders including my Tourette syndrome all the time. These communities have given me a lot of reassurance when I do feel this way however.

P20, 16 y, male

Some participants reported varying levels of support across different platforms:

Some are fantastic and make me feel better about myself every day. They help to relieve imposter syndrome and provide support daily. Not all communities offer the same support.

P21, 20 y, female

##### Finding Recognition

Being in OSCs allowed people with TDs to connect with likeminded others when offline options were not available*—*“There are no in person support groups in my area so without the online group, I would not have any contact with people like me” (P5, 39 y, male)—this was greatly valued and sometimes key to their mental health: “meeting people like me who completely understand was the key to my mental well-being, that no therapy could even come close to” (P33, 19 y, female). They described an increased understanding that they were not alone in living with a TD: “It’s good to know theres [sic] more than just me out there” (P1, 18 y, nonbinary). Participants were reassured that others encountered comparable struggles—“It’s just nice to have a space of like-minded people who have similar experiences to you” (P7, 17 y, nonbinary)—and had made similar choices regarding the management of their condition:

I felt empowered in this personal choice in Tourette’s [sic] communities as others have made this same decision for their own reasons, which has helped me feel not alone in making the right decision medically for myself and my Tourette’s.

P19, 22 y, female

##### Helping Others

Few participants described helping others directly, with many instead writing generally about the supportive and community nature of the OSCs. However, some spoke of giving back to others in their communities—“And in turn I’m able to help younger peers the same, and help them to understand that it’s okay to be different. Because I understand how scary it can be when you first develop tics” (P19, 22 y, female)—and supporting others whom they deem need it most:

So many people have had much rougher times of it. I try to help where I can, especially if someone doesn’t have the support network they need.

P11, 53 y, male

One respondent was an OSC moderator who saw helping others as part of this role:

As an admin, advocate, and mentor in the groups, I’m able to provide information to other members re [sic] where to access healthcare support.

P23, 52 y, nonbinary

##### Sharing Experiences

The process of sharing experiences was interwoven with several other empowerment processes and outcomes. This appeared particularly tied to relating to others’ shared experiences—“I like to analyse [sic] to a great extent and have people’s experiences I can relate to” (P3, 17 y, nonbinary)—or regarding the product or outcome of sharing experiences, rather than about this process directly. One example of this is how considering others’ experiences increased knowledge and understanding of how to manage tics:

Participating in online communities has allowed me to see what types of therapy and medications people used, and how they’ve learned to cope day to day with tics.

P37, 17 y, nonbinary

One participant shared concern about their tics being triggered by others’ sharing their experiences due to the influential nature of TDs:

I think that at the moment, the online support community has a negative impact because people’s tics are made worse by reading descriptions of other people’s tics.

P9, 18 y, female

Another commented regarding the negative comparison of their own experiences with others:

I sometimes feel jealous that so many people are in contact with doctors that have more of an understanding and bond with their patient so they’re getting somewhere positive and I’m stuck in the same place I’ve been for the past two years.

P30, 16 y, female

##### Changing Stereotypes and Stigma

An inductive finding from the data related to changes in attitudes and stigma through participation in OSCs. Being exposed to a range of lived experiences through OSCs positively challenged beliefs they held regarding the stereotypes of the condition:

Through use of communities such as discord I have gained a better understanding of the people my age living with tics. Ability to think beyond the stereotype of Tourette’s.

P21, 20 y, female

However, some perceived that their use of OSCs increased stigmatization from others to themselves and their community, which they reflected to be due to media coverage reporting links between social media and tics:

The impact that my online communities have on me is all positive, however I feel unable to talk to my healthcare professionals about this due to the recent ‘TikTok causes Tourette’s articles” [sic] (even though I know this is untrue).

P33, 19 y, female

This seems particularly salient in young people with TDs, with some respondents claiming this stigma is partly exacerbated by other OSCs users “faking” or misrepresenting their tic experiences in digital spaces:

I love the friends I have made but I’ve found that there are lots of kids who are too young to be on the app and they’re pretending more and more to be a part of our community so we face a lot of fake claiming because of them.

P2, 18 y, female

#### Empowering Outcomes Arising From OSCs

##### Being Better Informed

Most participants expressed that their knowledge of TDs has increased through OSC participation:

It made me feel more confident and knowledgeable about my Tourettes [sic] Syndrome.

P13, 18 y, female

They reported an increased understanding of the “correct” terminology to describe their condition and its related concepts:

I know the academical terms for everything related to my condition.

P4, 19 y, male

Additionally, they described increased awareness of treatment options available and strategies for self-managing tics to use when waiting to access treatment:

Online support communities have helped me gain a clearer understanding of some techniques available to manage tics, e.g [sic] CBIT [Comprehensive Behavioral Intervention for Tics]/Habit reversal, meaning I could work on these things whilst awaiting for professional treatment.

P28, 22 y, female

Some felt they had become more informed about living with TDs from the OSC than from HCPs:

I have learned more about Tourette syndrome through the online communities than through the health care system, which allows me to be better informed on a day to day basis living with it.

P39, 36 y, male

##### Feeling More Confident in the Relationship With HCPs

Through this aforementioned knowledge acquisition, participants also felt increased confidence in explaining their condition to HCPs, for reasons such as understanding of medical terminology:

Better knowledge of vocabulary to describe my experiences and symptoms in a way that is relevant to a medical professional.

P28, 22 y, female

Participants reported feeling more equipped for appointments, such as by preparing questions to ask HCPs, and feeling confident in interpreting the information given by HCPs:

I’ll know what to say, and what questions to ask. I’ll understand more of what i’m [sic] being diagnosed with.

P17, 16 y, other

This confidence was extended to their ability to advocate for themselves and potentially challenge and educate their HCPs regarding their care:

I was encouraged by members of online support groups to do my own research and to be my own advocate when speaking with healthcare workers.

P11, 53 y, male

Some participants reported that their OSC use had aided them in receiving an appropriate diagnosis. Many participants had not yet had an HCP appointment but felt that their OSCs had adequately prepared them for when they do and had given them the confidence to seek this help:

Being part of communities has given me the confidence to seek help regarding my tics. I have yet to have meaningful interaction with a doctor but have made an appointment to do so in the future, because I felt more equiped [sic].

P21, 20 y, female

##### Feeling Less Confident in the Relationship With HCPs

In contrast, many participants described a decreased level of confidence in their relationships with HCPs. Some felt their increasing knowledge led to negative judgment from HCPs:

I can better understand what they’re talking about but the more you’ve educated yourself on the topic the more judgemental [sic] doctors can get.

P2, 18 y, female

Participants also described how widespread negative health care experiences were shared on OSCs, such as HCPs disregarding their comfort, not being useful, not caring about the effects of treatments, or living with the condition and dismissing their concerns:

I’ve learnt how little drs [sic] know about tic disorders but they don’t seem to realize or care how that affects us when they are “treating” us. So many people with tic disorders often get dismissed even by the so called tic specialists.

P15, 17 y, nonbinary

Lack of access was also commonly described:

It is very much a common knowledge in the community that it is difficult to get a diagnosis and to find knowledgeable professionals who can help.

P8, 40 y, male

These negative perceptions and experiences that have been shared across their OSCs appear to have decreased their confidence in the health care system:

I would say that it has opened up my eyes to how badly people tics and Tourette’s get treated by the public/health care professionals.

P29, 17 y, nonbinary

Some participants expressed concerns about members being reliant on OSCs and peer support over HCPs, partly due to poor accessibility:

People use online communities instead of accessing appropriate healthcare (partly because they don’t see it as necessary and partly because there aren’t sufficient services).

P9, 18 y, female

Participants also shared concerns about disclosing to HCPs that they use OSCs. They reported that OSC use is stigmatized by HCPs, and that sharing this will increase judgment and accusations of “faking” their condition:

I feel as if telling them I am a part of the Tourette’s [sic] community on tiktok [sic] will make them doubt the legitimacy of my experiences, as I also have a diagnosis of FND [functional neurological disorder].

P33, 19 y, female

##### Enhanced Acceptance, Self-Esteem, and Mental Well-Being

Participants described how participation in OSCs helped to foster a sense of pride and build their confidence around their condition. This was regularly expressed through decisions to limit the suppression of their tics:

I also have started suppressing less in situations where doing so would make me uncomfortable/make my tics worse ... I’m more comfortable in allowing others to see me tic, and not being as embarassed [sic] as I was about it.

P7, 17 y, nonbinary

They often described feeling validated through their involvement, with this helping relieve feelings of imposterism:

Firstly it showed me that I do have tics and has shown me I don’t have to have complex tics all the time to still be valid as a ticker [sic].

P25, 19 y, female

They felt more able to be open about their condition and accept their diagnosis—“I don’t feel like a freak anymore, I didn’t even want to admit I had tics and the online community made me feel better about having them and being apart [sic] of the community” (P17, 16 y, other)—and increased confidence in their future living with tics:

It has helped me come to terms with the fact that I will be going through this for the rest of my life and it’s ok to live with it.

P8, 40 y, male

For some, this acceptance was linked to the decision not to pursue treatment:

Online support communities have helped me to accept my tics more so I have decided not to actively treat my Tourette syndrome with medication or behavioural [sic] therapy which has had a positive impact on my life.

P9, 18 y, female

Additionally, participants often attributed improved mental well-being to their use of OSCs:

I’m very, very glad I found these spaces early on after my tics started worsening. I think that if I hadn’t found these spaces, then I would be in a worse place mentally than I am now.

P7, 17 y, nonbinary

##### Feeling More Confident About Treatment Decisions

Many participants described feeling more confident about their treatment decisions, whether this be feeling more informed about treatment options and choosing to have treatment, or choosing not to pursue treatment to decrease tics and feeling confident in this decision. The group provided many members with information about treatments that allowed them to explore further options and take time to contemplate their decision:

In making me aware of treatment options, I have been able to weigh the pros and cons prior to meeting with healthcare providers thus keeping the decision from being an on the spot answer.

P5, 39 y, male

Support from others in the OSCs, and their shared experiences and opinions regarding treatment, increased their confidence in their decisions:

A lot of people automatically think we want to take medication when lots of us don’t. not because we like or want our tics but because the medication can have such a huge impact on not only your tics but you as a person, many of us don’t think it’s worth it.

P19, 22 y, female

Many perceived having treatment as a personal decision arising from individual circumstances:

We agree that there’s no “one size fits all” approach to treating tics as our experiences, where similar, also vary hugely. So it’s finding treatments and coping mechanisms that for [sic] right for you personally. [P10, 35 y, nonbinary)

A minority highlighted that they used the OSC to enhance their HCP’s guidance, rather than supplement or replace medical opinion:

I don’t let my online communities influence the desicions [sic] about my treatment made by healthcare professionals. However, if I read about a treatment that may help me I sometimes ask them about it and if they think it will help me.

P33, 19 y, female

Some participants, however, expressed a lack of confidence in treatment, partially due to widespread problems experienced and reported by others with the health care system:

I’ve come to realize other people within my community have had the same or similar issues involving the healthcare system. I feel as though my issues are something only I can take care of at this point.

P6, 18 y, male

##### Increased Control Over the Future

Participants described feeling more in control of their condition. They felt confident in making choices in the management of their TD, such as in their treatment decisions and in choosing not to suppress their tics:

Healthcare decisions ultimately lie with the individual. Not society, not the doctor. The person.

P11, 53 y, male

Participants reported gaining greater control in managing their condition by learning and using self-management strategies described within OSCs:

Online support communities have helped me to better deal with tic attacks, redirecting tics.

P20, 16 y, male

Some expressed preparation for hardships that may lie ahead of them. However, this was associated with a degree of decreased hope for some participants:

It made me aware of a lot of the hardships we can face before I ended up facing them myself. It has sometimes made me feel worse about my future, knowing the extent of the discrimination against us, but it’s also made me feel more prepared to face + work around it.

P7, 17 y, nonbinary

##### Enhanced Social Well-Being

Respondents overwhelmingly described a sense of community and belonging through their OSC participation:

It’s helped me become more self-accepting and has given me a stronger sense of belonging. I don’t feel isolated as I’m part of an empathetic community.

P23, 52 y, nonbinary

Participants described feeling less alone, which often went hand-in-hand with acceptance:

It has made me realise [sic] I’m not on my [sic] and I feel validated me.

P38, 48 y, female

Some participants described having formed new social relationships through OSCs, which appeared to be an important part in their journey in living with tics:

I have my own group of crazy amazing people and I don’t know what I would have done in the beginning with out [sic] them.

P30, 16 y, female

##### Raising Awareness

An inductive finding from the data suggested that a handful of participants felt OSCs had given users the opportunity and drive to talk about TDs and raise awareness of their condition: “It’s also made me more passionate about increasing awareness and education re TS and tic disorders” (P23, 52 y, nonbinary), both to help their peers—“but I trey [sic] feel amazing that I’m able to help so many people by talking and spreading awareness” (P30, 16 y, female)—and to work to change public perceptions of TDs: “I like to use my experiences to educate others on it which will make it easier to be ourselves in public” (P2, 18 y, female).

##### One Size Does Not Fit All

This inductive finding found that not all OSCs were regarded equally and varied in their setup, with participants voicing their concerns and wishes for more and better options. Numerous issues were raised; a primary problem appeared to be the perceived dominance of parents of children with TDs in many groups, which often led to feelings of ostracization in adults living with TDs:

The only downside I’ve experienced in online communities is that in communities with guardians of children and youth they can often be dismissive of adults with Tourette Syndrome as they do but [sic] want to acknowledge that they’re [sic] child(ren) may not outgrow the disorder, as doctors still tell parents is a possibility which they then cling to. This can lead to Adults with Tourette Syndrome bring [sic] ostracized to some degree.

P39, 36 y, male

One participant described parents requiring more support, which can be demanding for the participant:

Most members are parents who have children with Tourettes [sic], so I don’t feel I can find support as I end up too busy giving them support.

P36, 31 y, male

Additionally, one participant described how users with less “severe” tics do not use the OSCs as much as others:

We’ve found the ones who access (the most) it are mainly people who’s [sic] tics and TS are moderate to severe. We have a few members who’s [sic] tics are mild, but we find they don’t interact as much.

P10, 35 y, nonbinary

One participant also complained that some groups are not very active:

I wish a lot more of the disocord [sic] communities specifically were more active. And they all seem to have issues.

P19, 22 y, female

Participants reported preferring a variety of OSCs for tics to find the best fit for themselves:

So many of the groups out there cater to parents of kids with TS or to particular subsets, finding a good match wasn’t straightforward. I was overjoyed to eventually find a couple of groups that were more open and catered more to people with TS, regardless of age. I’m sure the groups I’m part of aren’t everyone’s cup of tea, either, so a broader set of communities to choose from would be a good thing.

P11, 53 y, male

## Discussion

### Principal Findings

This study aimed to explore empowerment-related processes and outcomes experienced by current users of OSCs for TDs, such as TS, expanding upon initial findings from research exploring experiences of using OSCs for TDs and social support mechanisms within them [32,51]. Thematic analysis of open-ended survey responses generated evidence of various empowerment processes and outcomes consistent with our empowerment framework [36], as well as additional inductive findings. Our findings align with previous research exploring OSCs for other LTCs such as HIV [47] and cancer [35], and how OSC use intertwines with health care experiences, group access, and systems [34], with this study generating findings specifically relating to those with TDs.

As in previous studies [32,51], many participants reported exchanging information as a key part of using OSCs for TDs and subsequently reported being better informed about many aspects of living with this LTC, such as improved understanding of treatment options and self-management of tics. Some participants reported learning more from their OSCs than through their health care systems. This reflects several studies highlighting patients’ experiences with HCPs in primary care: in the United Kingdom, young patients and their families have reported that their general practitioners provided little information about tics and tic management [59]. Patients and their families often have many concerns and questions about their tics [67]. Considering HCPs may not have sufficient answers, particularly regarding rarer conditions like TDs, it is understandable that individuals will seek help elsewhere [7,68,69], including from peers with experiential knowledge through similar health-related experiences.

This improved knowledge and understanding from OSC use was often applied to their interactions with HCPs, as participants reported increased confidence in interacting with HCPs within doctor-patient consultations, such as through knowing what questions to ask, the perceived appropriate language to use, and confidence to explain and advocate for their needs. This supports research illustrating that higher levels of patient empowerment are associated with greater participation in health care interactions [46,70] and contribute to the limited literature around the implications of peer digital support on relationships with HCPs [71]. However, although some participants reported feeling encouraged by their OSCs to seek help, within this study, there appeared to be a collective distrust of HCPs. This could potentially be a result of TDs being stigmatized within society and misunderstood by HCPs, as well as systematic barriers preventing health care access [14,72]. In this study, many participants shared their frustrations and experiences with health care systems. In contrast, those having positive HCP interactions or little health care difficulties may not share their perspectives in digital spaces. These adverse experiences echo previous research identifying obstacles in accessing primary care and specialist secondary care for tics [7,59]. Reading about others’ negative experiences and opinions and being confronted with negative aspects of their health condition and the health care system can be disempowering within OSCs [31,55], and some participants in this study did report feeling less confident in HCPs and in their treatment decisions. This nuanced pattern of the impacts of OSCs on health care interactions and empowerment contrasts with the more uniformly positive impact reported in broader health OSC studies [71]. Though increased empowerment has generally been shown to improve HCP relationships [34,36,37], in a survey of OSC users for a range of health conditions, Audrain-Pontevia and Menvielle [70] found that empowerment was negatively associated with their commitment to their relationship with their physician. Petrič et al [73] also demonstrated a complex relationship between OSC use and patient empowerment in relationships with HCPs, wherein exchanging information with users positively impacted self-efficacy but increased dysfunctional competencies in the relationship.

Some participants also reported concerns about sharing knowledge they had gained through OSCs with HCPs and disclosing their OSC use, due to perceived negative attitudes of HCPs around patient use of social media relating to TDs. Rupert et al [74] have found that HCPs often have negative reactions to patients sharing knowledge gained from OSCs. In regard to TDs, this could be due to HCP concerns around the quality and accuracy of web-based information [75], as well as potential stigma relating to recent publicity around links between functional tic-like behaviors and social media, particularly within the COVID-19 pandemic [76]. Increased presentations of tic-like behaviors [77,78] have been suggested to result from TD-related social media content, with some characterizing this rise as mass social media–induced psychogenic illness [76,79,80]. Other researchers have raised concerns with this theory, as it could perpetuate stereotypes that tics are voluntary attention-seeking behaviors, and add negative scrutiny toward people with TDs [81] who already express self-doubt over the legitimacy of their presentation [17,51]. This appears to be reflected in participants’ reported apprehensions about sharing their OSC use with HCPs. Mainly, women based in the United Kingdom shared these concerns, which may be due to media attention around concerns about social media and tics, particularly in the United Kingdom [82,83], and could also relate to gendered perceptions about tic presentation in women as being “attention-seeking” [17].

A key empowerment outcome from using OSCs was improved mental well-being, self-esteem, and acceptance of tics, which appeared to develop from access to emotional support and finding recognition from their peers. Similar to other studies around OSCs like mental health communities [84], OSCs were able to provide users with access to similar peers whom they often lack in their offline lives, helping reduce isolation and allowing them to form social connections. A previous thematic analysis of posts on one TD OSC demonstrated that people with TDs have reported concerns of “faking” their tics or that others believe so [51]. In this way, finding recognition and peer support was often seen as validating, as others understood their experiences and subsequently reduced their perceptions of being an “imposter.” This draws parallels with research surrounding peer support in those with another neurodevelopmental condition, attention-deficit/hyperactivity disorder, who also reportedly experience fears of imposterism that are helped by validation and acceptance received through OSCs [85]. This feeling may be more pronounced in TDs, as many features of tics—such as their ability to be suppressed and hidden, their wax-and-waning nature, and inconsistency in expression—may contribute toward these feelings of imposterism, as well as stigmatizing misperceptions of TDs in society [14]. Feelings of imposterism and inauthenticity have been reported by young women with TS, in questioning themselves on whether their tics are genuine or exaggerated—due to their ability to suppress tics, overlapping with symptoms of co-occurring conditions, and gendered perspectives in society around illness—and subsequently impacted on their sense of self and identity [17]. People with TDs often report feeling different, “weird” or “abnormal” from others [17,86,87], and interacting with peers who may have experienced similar “othered” experiences may assist in changing how they make sense of their condition and identity and empower them to take control over their condition [17,86,87]. Furthermore, one way those with TDs cope with the condition is how they align it with themselves, either integrating it within or externalizing it to their identity [88].

Interacting with similar others enhanced a sense of group identity, and OSCs were reported to increase self-acceptance and, in turn, influence how they managed their tics—including empowering them in making decisions relating to treatment, tic expression, and suppression. Through unmasking and not suppressing their tics, people with TDs may reduce pain related to tic suppression [9] and may contribute toward changing the social perceptions around visible differences, like tics, to create a more positive social environment for acceptance and social support to promote adaptive coping [88].

An empowerment outcome inductively identified from participants’ responses centered around raising awareness of TDs in society, to increase public understanding and decrease prejudice and discrimination toward people with TDs. This appears to map onto patient activism and collective empowerment [34]. Given that TDs are misunderstood and stigmatized in society [14], having a collective voice through OSCs might help in unifying marginalized individuals together and subsequently produce collective action to change societal attitudes. This patient activism could increase social change and improve service provision, although increased collective empowerment does not necessarily mean higher civic participation [44]. In interviews with people with an intellectual disability who used self-advocacy groups, Anderson and Bigby [89] reported that these groups helped increase their self-efficacy and confidence, including their ability to speak out for themselves and similar others, and to use their lived experiences as “expertise” in educating others about intellectual disability. As people with TDs may be another group marginalized and stigmatized within society, membership of TD groups could provide opportunities for creating individual and collective empowerment, which could counteract stigma and promote societal change.

Another inductive finding related to the suitability of different OSCs for different types of individuals using them, such as users who have tics or a TD themselves, or users who are parents or caregivers of children with TDs, with some users expressing difficulties with finding an appropriate group. This suggests that empowerment processes and outcomes can vary depending on the OSC used and its characteristics. A previous study into post–COVID-19 condition highlighted the size of groups and their platform as impacting sense of community and symptom management [90], and moderation was deemed to be important in gaining positive outcomes from OSC use for family caregivers [91]. In this study, the presence of carers appeared to affect the perceived value gained from the groups for people with TDs. The prominence of parent-dominated groups reported by participants related to feelings of exclusion and ostracization from these OSCs, supporting findings from Perkins et al [32]. Adults with TDs have reported limited options for general age-appropriate social support for TDs, with support being more available for children and parents or caregivers [17,22]. This variability in membership of OSCs for TDs can relate to disempowerment [31]: people with TDs and their families have some similar but also contrasting support needs. For example, parents of children with TS may feel anxious and stressed by their child’s symptoms and their impact on their daily life [92] and so require specific support for this. Therefore, it may be valuable to have distinct peer support options for people living with the condition and for those caring for them. Additionally, tailored support for subgroups could be important for addressing additional needs within specific groups—for example, having spaces for adults and women living with TDs [17,22].

### Strengths and Limitations

This study contributes to the limited amount of evidence exploring OSC use for TDs, and how it can be useful as part of TD management and coping with the long-term nature of TDs, as well as how empowerment manifests in those with TDs. The study was novel in the use of an established empowerment framework to guide analysis and in asking participants to share their experiences in their own words.

One limitation of this study was the size and nature of the sample. Despite a recruitment drive over 6 weeks across a range of platforms, the sample was relatively small. Future studies would benefit from a longer sampling period and working with further patient organizations to aid recruitment. The sample itself was also homogeneous in some respects. First, most participants were based in the United Kingdom; therefore, findings may relate to issues mainly within the UK health care systems. Second, participants were mainly female or nonbinary, meaning male views were less represented in our findings. This gender ratio contrasts with TDs being more prevalent in male patients [2]: this may be due to gender differences in using social support for health-related purposes [93], or potentially in coping with TDs [88]. Third, the age of participants in this study was skewed quite young. This could be reflective of the higher prevalence of TDs in younger adults [4,5]. Research concerning how age may affect OSC use, particularly in relation to empowerment, is limited. One study [94] suggested that digital support had a more pronounced effect on positive coping affect among older women with cancer compared to younger adults. A more age-diverse sample could reveal differing effects among age groups. Though not the aim of qualitative constructivist research [95], the small size and elements of homogeneity in the sample limit generalization, and findings should be interpreted with this consideration. Demographic and socioeconomic factors (eg, ethnicity and income), which could potentially impact on health care experiences, were also not recorded [96]. TD diagnosis was self-reported, with most participants reporting a diagnosis, although this was not verified, and standardized measures were not used to assess current tic symptomology.

A significant limitation of the study is that we sought to recruit only participants who were current users of OSCs, who were self-selected. Despite some participants reporting negative experiences [34], this introduced a bias toward identifying positive outcomes of OSCs, as more empowered people are more likely to continue using OSCs [97]. People who have left OSCs are presumably more likely to have had negative experiences or to have found the groups disempowering or of no value. These perspectives and experiences are therefore likely to have been underrepresented in our sample. Though this does not detract from the mostly positive and helpful experiences reported by participants in this study, which is corroborated by other research on OSCs [34-36,50], experiences of empowerment in people who leave OSCs should be investigated in further research to more thoroughly determine the usefulness of OSCs for people living with TDs.

In regard to analysis, data often appeared to overlap across several empowerment processes and outcomes. This partially reflects the difficulty in fully deciphering responses due to the text-based survey design, as authors could not ask participants to expand on their answers, but also illustrates the difficulties of using a previously defined framework for deductive analysis. It is difficult to concretely separate actions or processes from their results or outcomes. Processes are that where participants explained taking part in forms of empowerment, such as exchanging information, and the outcomes are the consequences of this, such as being better informed. These are likely to overlap as participants, for example, discuss learning more about TDs from communities, and authors had to make judgments about this in the analysis. Nonetheless, the framework provided a useful lens to understand empowerment experiences in OSCs, and to further understanding of empowerment in people with TDs in particular, which has had limited exploration. Furthermore, the inductively formed themes appeared to highlight more of the unique experience of living with a TD than deductively derived themes, which often highlighted findings more broadly applicable to OSCs, though this contributed to key work in this area by demonstrating the similarities between diverse health communities.

Finally, the open-ended questions used likely elicited more responses about empowerment outcomes, such as health care experiences and treatment decisions, rather than empowerment processes such as helping others. Our questions also did not explicitly ask participants about collective empowerment or disempowerment outcomes and processes they had experienced within OSCs, meaning there may have been additional findings relating to these aspects had this been explicitly asked about.

### Future Directions

A key perspective missing in research exploring OSCs for LTCs is from HCPs themselves: what do HCPs think about patients bringing knowledge and perspectives they have learned from OSCs? Participants in this study reported concerns about discussing information gathered in OSCs with their HCPs. Previous research suggests that HCPs often react negatively to patients sharing information from OSCs [74]. This may be more pronounced in interactions with patients with TDs, as within the COVID-19 pandemic, HCPs observed a marked increase in functional tic-like behaviors presenting in specialist services [77,78], leading to doubts about the legitimacy of patients presenting with tics [76,79-81]. The etiology and treatment of functional tic-like behaviors are different from tics within TDs, but can understandably appear similarly in patients with TDs, so there may be a mismatch in understanding between patients and HCPs. It is important to understand the barriers to transparency and knowledge sharing in doctor-patient consultations, which can jeopardize the doctor-patient relationship; breaking down these barriers has the potential to improve patient knowledge, empowerment, and self-management [74].

Future research should also consider the perspectives of different types of TD OSC users. As TDs typically have onset in childhood, caregivers of children with TDs may use OSCs [32]. The empowering or disempowering processes and outcomes they experience through OSC use may have similarities and differences from users with TDs; exploring these may illuminate ways in which these platforms can be used to support caregivers in both supporting themselves and their relative or friend with a TD. Additionally, the perspectives of moderators or administrators of OSCs should be considered as the gatekeepers who arbitrate over the digital space. In this study, one participant reported that they were a moderator of a TD OSC and emphasized the importance of their role in supporting other users. Previous research has shown this is an empowering process [98]. Future research should investigate the role of moderators in OSCs concerning empowerment more specifically. Finally, this study focused on people who were active users of OSCs for TDs—in contrast, another topic to explore is former users of these spaces and what led them to leave or disconnect from OSCs. As this study highlighted several disempowerment processes, it may be that disconnecting from OSCs could be a disempowerment outcome arising from participation.

This research was cross-sectional and qualitative, subsequently only providing a snapshot into the experience of interest. Therefore, it may be useful to experimentally investigate how using OSCs impacts people living with TDs over time, for example, by capturing changes in empowerment, clinical outcomes, and mechanisms such as shame, self-esteem, and self-management while using OSCs, particularly as there is a lack of experimental research in this area [99]. This could also help to identify the mechanisms of action of the effects of OSCs on empowerment [100]. Further research may provide evidence of the effects found in this study and contribute to the understanding of how OSCs may help people living with TDs to manage their condition.

### Conclusions

This study illustrates the empowering processes of OSC use and the empowering outcomes that their use can have on how people with TDs make sense of and live with long-term tics. This information could contribute to self-management guidelines concerning coping strategies and the importance of peer support in living with tics. It also emphasizes the relationship between OSC use and health care management and relationships with HCPs. HCPs must recognize their patients’ experiences of using OSCs and be aware of patients’ potential concerns about raising this with them.

## Data Availability

The datasets generated or analyzed during this study are not publicly available as they include sensitive data, but are available from the corresponding author on reasonable request.

## Appendix

Multimedia Appendix 1 Standards for Reporting Qualitative Research (SRQR) checklist.

Multimedia Appendix 2 Checklist for Reporting Results of Internet E-Surveys (CHERRIES).

Multimedia Appendix 3 Checklist for Reporting of Survey Studies (CROSS).

Multimedia Appendix 4 Coding framework based on empowering processes and outcomes questionnaires developed by van Uden-Kraan et al (2009).

## Abbreviations

CHERRIES: Checklist for Reporting Results of Internet E-Surveys
CROSS: Checklist for Reporting of Survey Studies
CTD: chronic tic disorder
HCP: health care professional
LTC: long-term condition
OSC: online support community
SRQR: Standards for Reporting Qualitative Research
TD: tic disorder
TS: Tourette syndrome
