# Supplementary material for: Exploring Empowerment in Online Support Communities for People Living With Tic Disorders and Tourette Syndrome: Qualitative Survey Study of User Experiences
Source: JMIR Form Res. 2025 Oct 9;9:e66912. doi: 10.2196/66912 (PMC12550454; doi:10.2196/66912)
Supplement: Multimedia Appendix 4 [file formative_v9i1e66912_app4.docx]

| **Codes** | | **Definitions** |
| --- | --- | --- |
| **Processes** | | |
|  | Exchanging information | Exchanging information and tips with other users. |
|  | Encountering emotional support | Other users being empathetic, complimentary, consoling and reassuring towards them. Having other users contact them privately or invite them to do so; express interest or pay particular attention to them in difficult times; offer them advice; point out their strengths; confide in them; ask for help and advice. |
|  | Finding recognition | Recognising themselves in other people's stories with others being an example to them. Feeling they are not the only one and that they are not so bad off. |
|  | Helping others | Being a positive example to other users, and/or offering advice and support to them. |
|  | Sharing experiences | Sharing experiences of their condition and everyday experiences with others. |
| **Outcomes** | | |
|  | Being better informed | Feeling better informed as someone with TD. Understanding their condition better, having a clearer picture of it and having more correct knowledge at their disposal to deal better with their condition. |
|  | Feeling more confident in the relationship with HCPs | Feeling prepared for HCP interactions, expressing knowledge of questions to ask, understanding of information provided by the HCP, and better ability to explain their needs. Expressing courage to raise matters and potentially oppose the HCP and think with them about treatment; feeling less dependent on them, being able to better judge when they need their help and having a changed relationship with them. |
|  | Improved acceptance of condition | They have improved acceptance of their condition and have/can give into the condition and have the ability to be more open about their condition, ask others for help and let them know when they cannot do something because of their condition. |
|  | Feeling more confident about treatment | Expressing ability to deal well with their condition and making the right decisions regarding their condition. As well as a better understanding of where to go with questions, and increased ability to stick to their treatment regime better and follow medical guidelines and advice from HCPs. |
|  | Increased optimism and control over the future | Expressing increased positivity and faith in the future; responsibility for their condition; being in charge of it and their lives, in control and able to influence it. |
|  | Enhanced self-esteem | Expressing a greater sense of worth, and a more positive attitude and contentedness towards themselves. |
|  | Enhanced social well-being | Feeling less lonely and making/having made new social contacts. |

**References**

van Uden-Kraan CF, Drossaert CHC, Taal E, Seydel ER, van de Laar MAFJ. Participation in online patient support groups endorses patients’ empowerment. Patient Education and Counseling 2009 Jan 1;74(1):61–69. doi: 10.1016/j.pec.2008.07.044
